# Viral metagenomics combined with metabolomics reveals the role of gut viruses in mouse model of depression

**DOI:** 10.3389/fmicb.2022.1046894

**Published:** 2022-11-15

**Authors:** Jiajia Duan, Wei Wang, Tao Jiang, Xiaoyang Bai, Chuanxin Liu

**Affiliations:** ^1^Department of Clinical Laboratory, The First Affiliated Hospital, College of Clinical Medicine of Henan University of Science and Technology, Luoyang, China; ^2^Department of Neurology, The Affiliated Hospital of Guizhou Medical University, Guiyang, Guizhou, China; ^3^Department of Medical Equipment, The First Affiliated Hospital, College of Clinical Medicine of Henan University of Science and Technology, Luoyang, China; ^4^Endocrine and Metabolic Disease Center, Medical Key Laboratory of Hereditary Rare Diseases of Henan, Luoyang Sub-Center of National Clinical Research Center for Metabolic Diseases, The First Affiliated Hospital, and College of Clinical Medicine of Henan University of Science and Technology, Luoyang, China

**Keywords:** gut viruses, viral metagenomics, neurotransmitters, depression, chronic restraint stress

## Abstract

Depression is a heterogeneous mental disorder that has been linked to disturbances in the gut microbiome. As an essential part of the gut microbiome, gut virome may play critical roles in disease progression and development. However, the relationship between the effect of gut virome on neurotransmitter metabolism and depression is unknown. We evaluated the alterations of gut virome and neurotransmitters in chronic restraint stress (CRS)-induced mouse model of depression based on viral metagenomics and LC–MS/MS metabolomics analyses. The results reveal that the gut virome profile of CRS group differed significantly from CON group. *Microviridae* was the most abundant differential viral family in both groups, followed by *Podoviridae*, while *Siphoviridae* was only enriched in CRS group of the top 100 differential viruses. The differential viruses that predicted to *Enterobacteriaceae* phage, *Gammaproteobacteria* phage and *Campylobacteraceae* phage were enriched in CRS group. Furthermore, 12 differential neurotransmitters primarily involved in the tryptophan metabolism pathway were altered in depressive-like mice. Besides, tryptamine and 5-methoxytryptamine hydrochloride were strongly associated with differential viruses belonging to *Podoviridae* and *Microviridae*. Our findings provide new insight into understanding the potential role of the gut virome and metabolites in depression.

## Introduction

Depression is a common psychiatric disorder that imposes an enormous socio-economic loss worldwide. About half of the world’s population suffers from depression at some point in time, with an average episode lasting about 6 months (Calliope Holingue, 2018). A prospective epidemiological study reported a lifetime prevalence of major depressive disorder as high as 30–40% (Moffitt et al., 2010). Moreover, depression has been linked to various chronic physical diseases, including cardiovascular diseases, obesity, diabetes, hypertension, cancer, cognitive impairment, chronic respiratory disease, and others (Gold et al., 2020). The most widely accepted pathogenesis of depression includes genetic, psychosocial, and biological factors. Biological factors include theories about monoamine neurotransmitters, neuroplasticity, neurogenesis, inflammation, and circadian rhythm (Peng et al., 2015; Mendoza, 2019). However, the exact biological mechanisms remain unknown.

Recently, multiple studies have shown that the pathogenesis and treatment of depression are inseparable from the gut microbiome (Valles-Colomer et al., 2019; Zheng et al., 2021). Gut microbiota can be influenced by the host’s genetic makeup, diet, and environment. Gut bacteria can produce various neuroactive substances, including gamma-aminobutyric acid (GABA) and serotonin, which are involved in the development of various psychiatric disorders through the gut-brain axis (Dalile et al., 2019; Agirman et al., 2021). Furthermore, animal studies have shown that depressed phenotypes can be transferred to non-depressed animals through microbiome transplantation (Zheng et al., 2016; Zhang et al., 2019). As part of the gut microbiome, the potential roles of the gut virome, such as maintaining homeostasis and promoting disease progression, have also been investigated. Although the cause remains unknown, there are evidence for an association between the virome and human diseases, like SARS-CoV-2 infection, human immunodeficiency virus infection, inflammatory bowel disease, diabetes, obesity, and high blood pressure (Bai et al., 2022). Viruses, especially bacteriophages, are gradually gaining researchers’ attention. A recent study confirmed that bacteriophage infection of a cytolysin-producing strain of *Enterococcus faecalis* reduced liver disease severity in patients with alcoholic hepatitis (Duan et al., 2019). However, little is known about the association between gut virome and depression. Researchers have previously characterized the signatures of gut bacteriophages using a cross-sectional whole-genome shotgun metagenomics analysis of fecal samples from major depressive disorder patients (Yang et al., 2020b). However, because metagenomics contains the genetic information of an entire microbial community, it is difficult to capture as much virus information as possible.

Viral metagenomics is a new field of study that combines the theory of metagenomics with the existing molecular biology virus detection technology. Viral metagenomic pipeline comprises sample collection, processing, sequencing, and bioinformatics analysis (Rose et al., 2016; Nooij et al., 2018; Cantalupo and Pipas, 2019; Santiago-Rodriguez and Hollister, 2020). The critical step of viral metagenomics is the enrichment of virus-like particles (VLPs) from samples containing a large number of microbial cells. Viral metagenomics provides a more comprehensive understanding of the alterations in virus composition and functions between the disease and the physiological state of the host. Given the high correlation between the gut microbiome and body metabolism, metabolomics has become a powerful tool for studying the impact of virome on host health and disease (Yadav et al., 2016; Yang et al., 2020b). To the best of our knowledge, no study has reported whether the gut virome can affect neurotransmitter metabolism associated with depressive-like behaviors. We developed a mouse model of depression using CRS in this study. We aimed to investigate the association between gut virome and neurotransmitters in a mouse model of depression by combining viral metagenomics and liquid chromatography tandem mass spectrometry (LC–MS/MS) metabolomics analysis. Our findings will provide new insights into the possible role of the gut virome in depression.

## Methods and materials

### Animal experiments

Male adult C57BL/6 J mice aged 6–8 weeks were used in the present study. All mice were housed under relatively stable conditions with a 12 h light–dark cycle, 55 ± 5% humidity, a constant temperature of 23 ± 2°C, and allowed water and food *ad libitum*. The animals were randomly divided into two groups (random number table method). The mice in the control group were housed 3–4 per cage, while those in CRS group were housed individually. The body weights of mice were measured at the baseline and once a week during the experiment. All mice were purchased from Beijing Weitong Lihua Biotechnology Co., Ltd. All animal experiments complied with ethical regulations and were approved by the Animal Care Welfare Committee of the GuiZhou Medical University.

CRS group mice were subjected to the chronic restraint stress protocol for 21 days, 6 h per day. The mice were placed in a 50 ml centrifuge tube used as a restraint tube (the centrifuge tube was scalded in advance with air holes, evenly dispersed on the wall of the centrifuge tube, and there was a small hole in the middle of the lid to allow the mouse’s tail to pass through). CRS group mice were deprived of water and food during restraint, while the mice in the control group were housed under relatively stable conditions.

### Behavioral assays

Behavioral analyses were performed by individuals blinded to experimental conditions. The experiments were conducted in a soundproof room. The behavior of all animals was recorded and analyzed by SuperFst/Tst Video Tracking Software.

### Sucrose preference test

The sucrose preference test includes an adaptation training phase and a testing phase. Mice were housed individually and habituated to water and 1% sucrose solution for 2 days during the adaptation training phase. The bottles were rotated daily to avoid positional preferences. Mice were then deprived of food and water for 12 h. In the testing phase, mice were exposed to two bottles containing 1% sucrose solution and tap water for 12 h in the dark. Two bottles were removed and weighed, the sucrose solution and water consumption were recorded, and the sucrose preference ratio was calculated.

### Forced swimming test

The mice were placed in a transparent cylindrical container (pool, 30 cm in height and 15 cm in diameter) with a water depth of 15 cm and temperature of 24 ± 1°C. The time of swimming and immobility state of mice during the 5 min test phase was recorded, and the immobility ratio was calculated. Swimming behavior refers to the mice swimming on and around the pool level, whereas immobility behavior refers to mice having no other behaviors except an upward movement to avoid drowning.

### Tail suspension test

The rear one-third of the mouse’s tail was taped and hung from a special bracket. The mouse head was 15 cm away from the bottom surface, with a white background creating a stark contrast with the mouse fur. The struggling and immobility states of the mice were recorded and calculated after 5 min. Immobility was defined as the mice moving only their forelimbs and not their hind limbs.

### Viral DNA extraction and sequencing

VLPs from fecal samples of mice were enriched and purified. Fecal samples were collected and grounded, and a pre-chilled Extraction Buffer (EB) was added. The precipitates were removed after vortexing and centrifugation. The supernatant was filtered through a 0.45 μm filter membrane to remove cells and fragments and added to Precipitation Buffer (PB). The supernatant was discarded after standing at 4°C for 2 h, and the precipitate was resuspended in 200 μl EB. The enzyme mix buffer, enzyme mix, and stop solution were sequentially added to the reaction mixture and incubated at 65–70°C for 10 min. After centrifuging at 2000 rpm for 5 min, 200 μl supernatant was stored at −20°C for use in subsequent experiments. Viral DNA and RNA were extracted with Magen R6662-02 MagPure Viral DNA/RNA Mini LQ Kit according to the manufacturer’s instructions. Whole-genome amplification was performed using Qiagen 150,054 REPLI-g Cell WGA & WTA Kit (Qiagen, Dusseldorf, Germany). Qualified samples were used for library construction using NEB Next® Ultra II™ DNA Library Prep Kit for Illumina (NEB, USA). After quality inspection of the library with the Qubit® dsDNA HS Assay Kit (Life Technologies, USA) and the Agilent 4,200 TapeStation (Agilent, USA), the Illumina Novaseq 6,000 was used for sequencing with 150 bp paired-end mode.

### Bioinformatics analysis of the virome

Trimmomatic (v0.36) was used to obtain high-quality data by removing low-quality raw data (Bolger et al., 2014). The clean reads were aligned with the Silva.132 database and the mouse database using the BWA software (Li and Durbin, 2009). The alignments with coverage below 80% of the sequence length were filtered out, and host genomic sequences were eliminated. The clean reads were then aligned with the Virus-NT database for the initial classification. Clean data were assembled using Megahit software (Li et al., 2016). Contigs from all samples were clustered using CDHIT.

The CheckV software (Nayfach et al., 2021) was used to predict the set of potential virus sequences within the assembled sequence. According to the alignment of virus contigs and the virus-NT database blast (v2.9.0+), the best hits with e < 1e-5 were selected for annotation to obtain functional information about the virus. A CRISPR-Cas spacer database was constructed from bacterial genomes in the Refseq database using the CRISPR Recognition Tool (CRT, http://www.room220.com/crt/). The contigs identified above were then aligned by blastn-short (v2.9.0+), satisfying e-value ≤1e-10, alignment similarity ≥95%, and spacer coverage of 80%. The best hit was chosen as possible host information for phage. Only a subset of viral contigs can predict possible hosts due to the limited number of known spacer sequences.

### Neurotransmitters analysis by LC–MS/MS

After thawing and crushing the fecal samples, 0.05 g of the sample was mixed with 500 μl of 70% methanol/water. The sample was vortexed at 2500 r/min for 3 min and centrifuged at 12,000 r/min for 10 min at 4°C. About 300 μl of supernatant was transferred to a new centrifuge tube and refrigerated at −20°C for 30 min. The supernatant was then centrifuged at 12,000 r/min for 10 min at 4°C, and 200 μl of supernatant was separated for further LC–MS analysis.

The sample extracts were analyzed using an LC-ESI-MS/MS system (UPLC, ExionLC AD, https://sciex.com.cn/; MS, QTRAP® 6,500+ System, https://sciex.com). The analytical conditions were as follows:

HPLC column, Waters ACQUITY UPLC HSS T3 C18 (100 × 2.1 mm i.d.，1.8 μm); solvent system, water with 0.1% formic acid (A), acetonitrile with 0.1% formic acid (B); the gradient was initiated at 5% B (0 min), increased to 95% B (0–8 min), 95% B (8–9.5 min), and then decreased to 5% B (9.6–12 min); flow rate: 0.35 ml/min; temperature: 40°C; injection volume: 2 μl.

AB 6500+ QTRAP® LC–MS/MS System, equipped with an ESI Turbo Ion-Spray interface, operating in positive and negative ion modes, and controlled by Analyst 1.6 software (AB Sciex). The ESI source operation parameters were as follows: an ion source, turbo spray, source temperature 550°C, ion spray voltage (IS) 5,500 V (Positive), −4,500 V (Negative); curtain gas (CUR) was set to 35.0 psi; and DP and CE were further optimized for individual MRM transitions. A specific set of MRM transitions was monitored for each period according to the neurotransmitters eluted during this period.

### Statistical analysis

All statistical analyses were performed using R (v3.5.1) and SPSS 26.0. Alpha and beta diversity analysis was performed using the phyloseq package. Beta diversity was analyzed using principal coordinate analysis (PCoA) with Bray-Curtis distance, and the statistical significance of the clustering pattern in ordination plots was evaluated using permutational ANOVA (PERMANOVA). Data from behavioral results and body weight were compared using Student’s t-test, while the virome and metabolites data were compared using Wilcoxon rank-sum test. Correlation between relative abundances of differential viruses and between differential viruses and metabolites was determined by spearman correlation analysis. GraphPad Prism 9.3.1, Origin 9.8.0, and Cytoscape 3.8.0 were used for graphing.

## Results

### CRS-induced depression-like behaviors in mice

**Figure 1:**
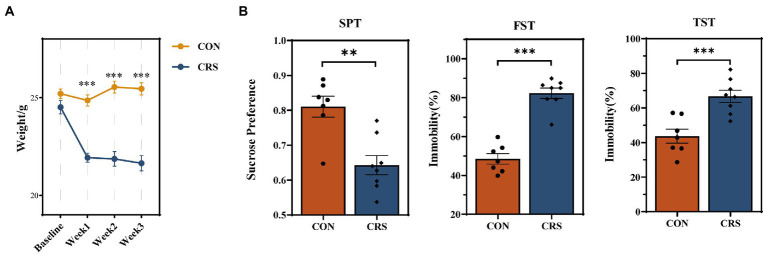
Behavioral results and body weight changes in mice during the experiment. **(A)** Weight changes per week from baseline to end of the study. **(B)** Behavioral results of sucrose preference test (SPT), forced swimming test (FST), and tail suspension test (TST). The data are represented as the mean ± standard errors of the means (SEM). **p < 0.01, ***p < 0.001.

In the present study, mice were exposed to CRS for 21 days to induce depression-like behaviors. The depression-like behaviors in mice were evaluated by the sucrose preference test (SPT), forced swimming test (FST), and tail suspension test (TST). The baseline and weekly body weights of mice were measured during the experiment. The body weights of mice in CRS group (N = 8) were considerably lower than those in CON group (N = 7) after 3 weeks of stress (*p* < 0.001, Figure 1A). SPT, TST, and FST were examined at baseline in both groups of mice and no difference between the two groups was observed (Supplementary Table S1). Significant reduction in sucrose consumption in SPT (CON: 0.811 ± 0.030 [mean ± SEM]; CRS: 0.643 ± 0.027 [mean ± SEM]; *p* = 0.001), increase in immobility ratio in FST (CON: 48.487 ± 2.718 [mean ± SEM]; CRS: 82.289 ± 2.672 [mean ± SEM]; *p* < 0.001) and increase in immobility ratio in TST (CON: 43.671 ± 4.028[mean ± SEM]; CRS: 66.709 ± 3.535 [mean ± SEM]; *p* < 0.001) were observed in CRS group compared to the control group (Figure 1B). These results indicated that CRS had induced depression-like behaviors in mice.

### Diversity and composition of the virome community between CON and CRS groups

**Figure 2:**
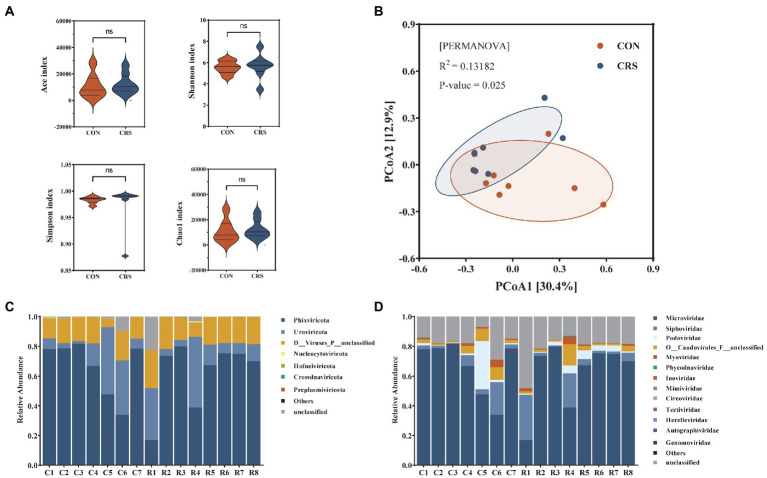
Comparison of viral community diversity and composition at contigs level between the two groups. **(A)** Alpha-diversity presented by Ace, Shannon, Simpson, and Chao1 index. No significant differences were detected. **(B)** Beta diversity visualized using a PCoA plot with Bray–Curtis dissimilarity distances. Compositions of the viral communities at the phylum level **(C)** and family level **(D)**.

DNA virome in the feces of CRS and CON groups was analyzed. Alpha diversity was analyzed to assess differences in subject-to-subject diversity. The alpha-diversity analysis showed no significant difference in gut virome composition of the two groups (ACE index, *p* = 0.536; Shannon index, *p* = 0.694; Simpson index, *p* = 0.121; Chao1 index, p = 0.536; Figure 2A). Meanwhile, Beta diversity was analyzed to measure group-to-group diversity. β-diversity analysis based on PCoA with Bray-Curtis distance showed significant differences between the two groups (R^2^ = 0.132, *p* = 0.025, PERMANOVA; Figure 2B).

We identified and taxonomically annotated the viral sequences to clarify the virome characteristics between the two groups. From all samples, 84,108 contigs were obtained. Among them, phage and non-phage viruses accounted for 89.48 and 10.52%, respectively, and the predominant viral genome type is dsDNA (87.64%) (Supplementary Figure S1A). Viral community compositions at the phylum and family levels are shown in Figures 2C,D, and Supplementary Figure S1B. The five most prevalent virome phyla detected in all samples were *Phixviricota*, *Uroviricota*, *Nucleocytoviricota*, *Hofneiviricota*, and *Cressdnaviricota* (Figure 2C). The top 10 virome families were *Microviridae*, *Siphoviridae*, *Podoviridae*, *O__Caudovirales_F__unclassified*, *Myoviridae*, *Phycodnaviridae*, *Inoviridae*, *Mimiviridae*, *Circoviridae*, *Tectiviridae* (Figure 2D). We performed gene set functional enrichment in all samples using KEGG pathway analysis. Genetic information processing was the predominant enrichment pathway in KEGG level 2 and DNA replication proteins in KEGG level 3 (Supplementary Figures S2A,B).

### The differential profile and relationship of the virome between two groups

**Figure 3:**
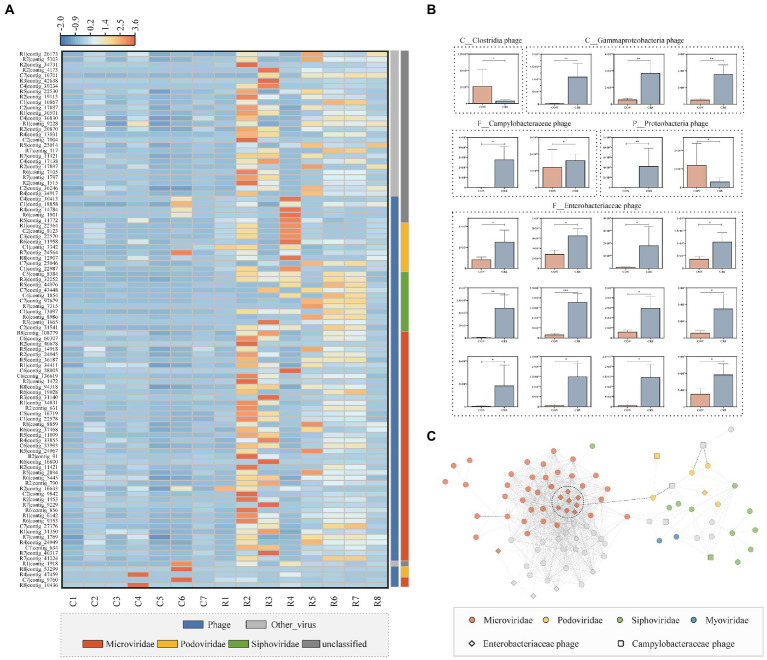
Differential viral profile and the correlations between the differential virus contigs of the two groups. **(A)** Heatmap of the differential virus contigs (the top 100 of relative abundance); the color scale bar shows z-score values after z-score row normalization. **(B)** Bar plot of 20 differential viral contigs, of which the potential bacterial hosts were predicted. The data are represented as the mean ± standard errors of the means (SEM). *p < 0.05, **p < 0.01, ***p <0.001. **(C)** The differential virome co-expression network with Spearman’s rank correlation coefficient (|r|>0.6, *p* < 0.01). Dots with different colors indicate different families.

We compared the virome composition at the contigs level to gain a deeper understanding of the differential virome profile between CON and CRS groups. A total of 5,006 contigs were differentially enriched in the two groups. We have analyzed the top 100 viral contigs with the highest relative abundance (Figure 3A). Among the top 100 contigs, 95% (95/100) were enriched in CRS group. *Microviridae* was the most abundant differential family, followed by *Podoviridae* in CON and CRS groups. However, *Siphoviridae* was only enriched in CRS group. Figure 3B shows the comparison of the relative abundance of 20 contigs that were successfully annotated to the family level of the virome and predicted to the possible bacterial hosts between CON and CRS groups. The contigs predicted to be the phages of *Enterobacteriaceae*, *Gammaproteobacteria*, and *Campylobacteraceae* were enriched in CRS group. Furthermore, two contigs, including *Clostridia* and *Proteobacteria* phages, were enriched or partially enriched in CON group. Co-expression network of the differential virome was constructed to determine the relationship within the differential virome (|r|>0.6, *p* < 0.01; Figure 3C). We discovered that members of *Microviridae* had a strong relationship within the cluster, with the majority exhibiting a positive correlation. R2|contig_16633 served as the link between *Microviridae* cluster and *Siphoviridae*, *Myociridae*, *Podoviridae*, and other unclassified viral clusters. The viruses belonging to *Podoviridae* showed a weak correlation with each other. Notably, C7|contig_43448, *Podoviridae* member with a negative correlation with *Microviridae* cluster.

### Disturbances of fecal metabolic signatures between CON and CRS groups

**Figure 4:**
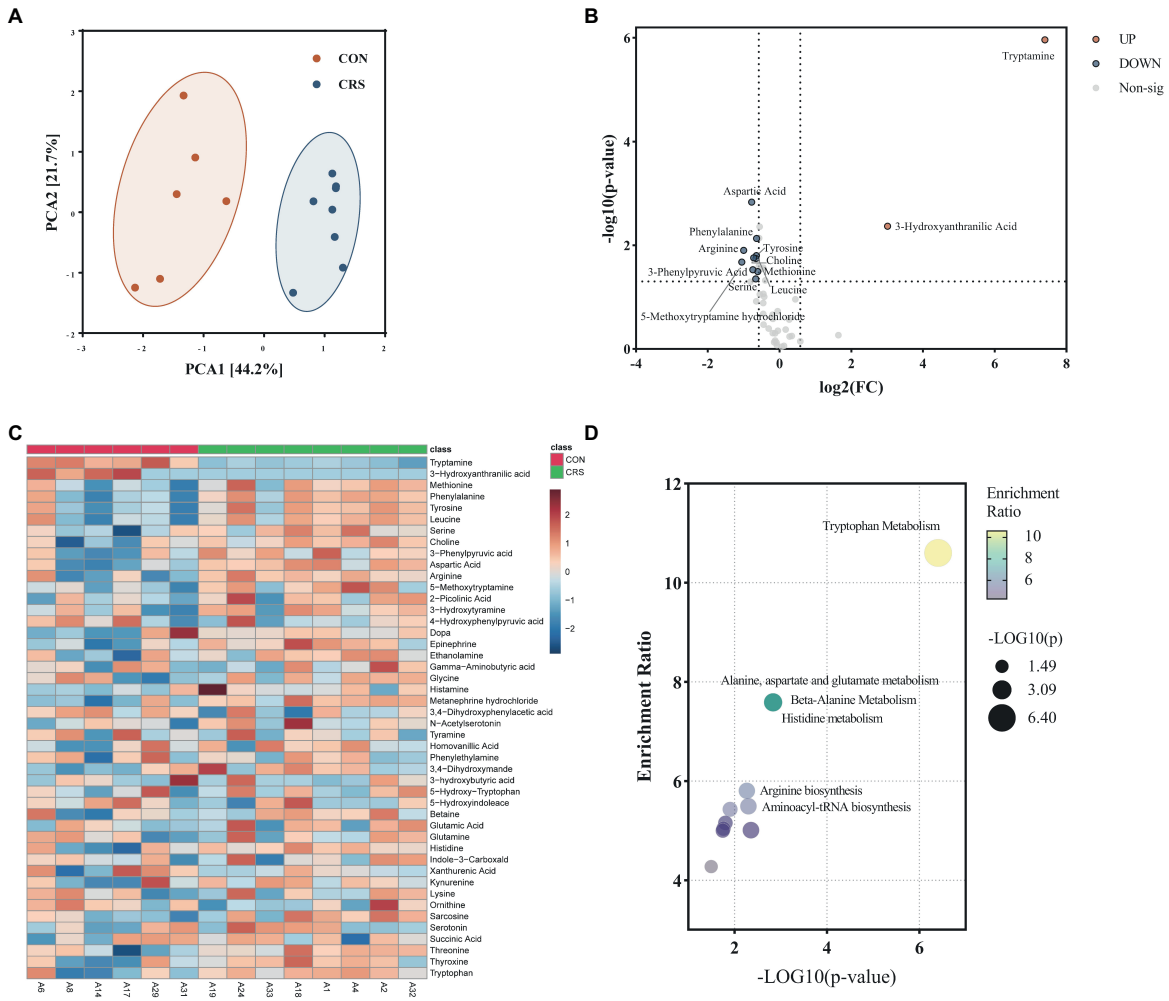
Characterization of metabolites profile of neurotransmitters from mice fecal. **(A)** Principal component analysis (PCA) Scores plot between the two groups. The explained variances are shown in brackets. **(B)** Volcano plot for differential metabolites. Significantly regulated metabolites between groups determined by fold change and value of p (FC>|1.5|, *p* < 0.05). Red dots represent increased metabolites in CON group; blue dots represent decreased metabolites in CON group. **(C)** Heatmap of all neurotransmitters; differential metabolites were indicated by an asterisk. **(D)** Pathway enrichment analysis of differential metabolites.

LC–MS/MS was used to assess the metabolic profiles of CON and CRS fecal samples. A total of 55 neurotransmitter metabolites were identified in the present study. Metabolites with missing values greater than 60% per group were eliminated after data processing, leaving 46 neurotransmitter metabolites for further analysis. Principal component analysis (PCA) was performed to reduce dimension and identify specific metabolic features that drive group separation. One outlier sample was removed for further analysis. PCA score plot showed a significant distinction between the two groups (Figure 4A). A volcano plot with Fold change (FC)>1.5 and value of p <0.05 was applied to identify the specific metabolites differences between the two groups (Figure 4B). The two groups differed in 12 metabolites. The CON-enriched metabolites included tryptamine and 3-hydroxyanthranilic acid. Meanwhile, CRS-enriched metabolites included methionine, phenylalanine, tyrosine, leucine, serine, choline, 3-phenylpyruvic acid, aspartic acid, arginine, and 5-methoxytryptamine hydrochloride. Furthermore, all signatures of 46 neurotransmitter metabolites between the two groups are shown in Figure 4C. Quantitative enrichment analysis (QEA) of differential metabolites was performed using MetaboAnalyst 5.0. Three of differential metabolites were mainly involved in tryptophan metabolism (5-methoxytryptamine hydrochloride, tryptamine, and 3-hydroxyanthranilic acid), and six were involved in aminoacyl-tRNA biosynthesis (phenylalanine, arginine, aspartic acid, methionine, leucine, and tyrosine) (Figure 4D). Analyses of neurotransmitter metabolites revealed a divergent profile between the mouse model of depression and controls; tryptophan metabolism and aminoacyl-tRNA biosynthesis might be related to these differences.

### Co-occurrence network analysis between the gut virome and neurotransmitter metabolites in CON and CRS groups

**Figure 5:**
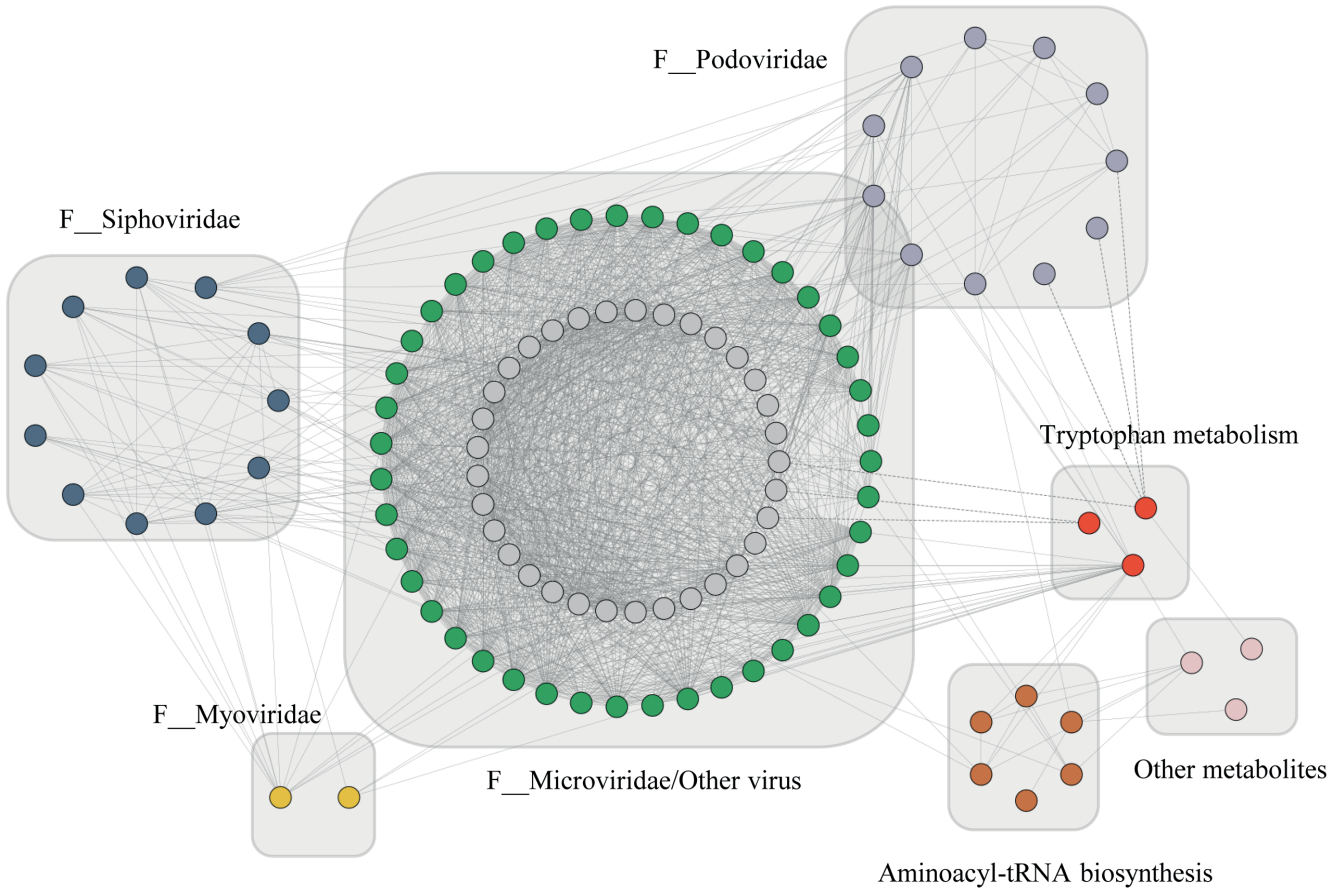
Co-occurrence network between the differential virome and metabolites with Spearman’s rank correlation coefficient (|r|>0.6, *p* < 0.01). Viruses were shown in blue, green, yellow, purple, and gray dots and metabolites were shown in red, orange, and pink. Solid lines indicate positive correlations, and dashed lines represent negative correlations.

A co-occurrence network was performed based on Spearman’s rank correlation analysis (|r|>0.6, *p* < 0.01), to explore the association between differential gut virome and neurotransmitter metabolism in mouse models of depression and controls (Figure 5). We discovered that tryptamine, enriched in the tryptophan metabolism pathway, was negatively correlated with three *Podoviridae* viruses. 5-Methoxytryptamine hydrochloride demonstrated a strong positive correlation with 13 *Microviridae* viruses and four *Podoviridae* viruses. A positive correlation was observed between aminoacyl-tRNA biosynthesis pathway metabolites and gut virome. Arginine was positively correlated with two viruses of *Microviridae* and one of *Siphoviridae*, while methionine was positively correlated to one *Siphoviridae* virus. However, two metabolites, phenylalanine and choline showed no significant correlation with the viruses. These results indicate that altered gut virome and neurotransmitter metabolites formed a synergistic and node-related co-occurrence network between CON and CRS groups, in which metabolites enriched in the tryptophan metabolism pathway played a potential role.

## Discussion

The microbiome is the microorganisms found in mammalian hosts, including viruses, bacteria, archaea, fungi, and protozoa. Recently, microbiome research has expanded in number and scope. Since the coronavirus disease 2019 (COVID-19) pandemic, researchers’ interest in virome has increased tremendously. The gut has been shown to contain a diverse virome. Several studies have summarized the interaction between gut virome and human diseases, such as diabetes mellitus, asthma or pneumonia, and hypertension (Han et al., 2018; Ma et al., 2018; Romero-Espinoza et al., 2018; Cinek et al., 2021). However, it has not been established if the gut virome is involved in neurotransmitter metabolism to mediate the occurrence of depression. We constructed a CRS depression mouse model and investigated the association between gut virome and neurotransmitter metabolites using viral metagenomics and LC–MS/MS methods to provide insights into the role of the gut virome in depression.

In the present study, we found that body weight of mice was reduced after 1 week of CRS. The depressive symptoms, including decreased sucrose preference ratio and increased immobility time in FST and TST, were shown in mice of CRS group. We then combined the α-diversity and β-diversity analyses with species composition to evaluate the diversity and compositional differences of virus communities within the dataset. Analysis of viral α-diversity (Ace, Shannon, Simpson, and Chao1 indices) revealed no significant difference between CRS and CON groups, indicating that the diversity within each fecal sample was relatively stable between the two groups. However, β-diversity analysis based on Bray–Curtis dissimilarity distances showed that CRS group could be distinguished from CON group. The results indicated that CRS could affect the gut virome in mouse models of depression. It contradicted a previous human study that found no significant global alteration of gut virome between major depressive disorder patients and healthy controls (Yang et al., 2020b). This may be attributable to the small sample size of our study or the potential difference between animals and humans.

Moreover, we have described the phylum and family-level virus composition of the two groups. *Phixviricota* was the most abundant phylum in both groups, followed by *Uroviricota*. The top five most abundant families in both groups include *Microviridae*, *Siphoviridae*, *Podoviridae*, *Myoviridae*, and *Phycodnaviridae*. Multiple studies showed that the human gut virome mainly comprises tailed, dsDNA viruses from *Caudovirales* and non-tailed, ssDNA viruses from *Microviridae* (Reyes et al., 2012; Aggarwala et al., 2017; Mirzaei and Maurice, 2017; Shkoporov and Hill, 2019). The most important members of *Caudovirales* include *Myoviridae*, *Podoviridae*, and *Siphoviridae* (Liang and Bushman, 2021), which is consistent with our findings. These results indicate that the major components of the virome in mouse and human studies are similar. Since mice are not affected by various human-related environmental, dietary, and social factors, they can more accurately reflect changes in the gut virome in response to different situations.

We compared the viral profiles of CON and CRS groups at the contig level to investigate whether a specific virus cluster differed between the two groups. Interestingly, the majority (95%) of the top 100 relative abundant differential virus contigs were enriched in the CRS group. The most enriched differential taxa in the CRS group were *Microviridae*. Some *Microviridae* members have been identified as the prophages of *Bacteroides* and *Parabacteroides* species (Krupovic and Forterre, 2011). Since *Bacteroides* were confirmed to be enriched in the gut microbiome of depressed patients (Yang et al., 2020b), our results suggested that *Microviridae* may be associated with depression by influencing the dysregulation of *Bacteroides.* Moreover, *Microviridae* members exhibit many internal correlations, which may help restore dysregulated community states to healthy homeostasis (Manrique et al., 2017).

Most gut viromes are bacteriophages [32], and their role in gut physiology may be far more important than altering bacterial communities by bacteriophage infection (Miedzybrodzki et al., 2008; Ksendzovsky et al., 2012; Virgin, 2014). Changes in the gut phage community composition may contribute to the relevant transition between health and disease. In our study, the most abundant differential viral taxa between CRS and CON groups were identified, and they all belonged to bacteriophages. Their presumed bacterial hosts were mainly *Enterobacteriaceae*, followed by *Gammaproteobacteria*. Most *Enterobacteriaceae* and *Gammaproteobacteria* phages were enriched in CRS group. A previous study reported an increase in *Enterobacteriaceae* prevalence in patients with the major depressive disorder (Jiang et al., 2015). The *Enterobacteriaceae* family includes several inflammogenic enteric pathogens that could be translocated into the systemic circulation in depressed patients due to the increased permeability of the gut wall (O'Malley et al., 2010). Some gut phages are lytic and can influence the host cell transcription or translation process to produce more phage components before lysing the host cell membrane and releasing the phage particle into the local environment. Most gut phages are lysogenic, implying that they introduce their DNA into the host cell and replicate passively with their host over time without producing virions (Cryan et al., 2019). Research showed that *Enterobacter* phages prevalent in COVID-19 patients may be involved in the host immune response (Zuo et al., 2021). Enrichment of *Enterobacteriaceae* phages in CRS group of our study suggested that this phage might play an important role in depression. However, further research is required to determine whether the phages are lysogenic. If the causal relationship between *Enterobacteriaceae* and depression were clarified by future studies, the enriched *Enterobacteriaceae* phages in mouse models of depression would provide resources and directions for the application of phage therapy.

We then used targeted metabolomics to investigate neurotransmitter differences and the potential relationship between neurotransmitters and gut virome. We discovered 12 metabolites that changed after CRS procedure, most of which were involved in the tryptophan metabolism pathway and the aminoacyl-tRNA biosynthesis pathway. Tryptamine is the indoleamine metabolite of the essential amino acid tryptophan (Jenkins et al., 2016), and multiple studies have revealed that tryptamine can regulate dopaminergic, serotonergic, and glutamatergic systems (Khan and Nawaz, 2016; Berry et al., 2017). Tryptophan in the diet can be converted into tryptamine in the gut by symbiotic bacteria, which further activates 5-HT_4_ receptors to regulate gastrointestinal motility (Jenkins et al., 2016; Bhattarai et al., 2018). Furthermore, synthetic modifications of tryptamine can produce serotonin and melatonin. In the current study, we discovered that tryptamine was significantly reduced in CRS group, while 5-methoxytryptamine hydrochloride was increased. Since serotonin is essential for maintaining a normal emotional and psychological state in patients with depression, the decrease in tryptamine indicated a possible serotonin deficiency related to depression-like behaviors in CRS group. Another significant metabolic pathway was aminoacyl-tRNA biosynthesis pathway, which was an important metabolic pathway of protein synthesis. In addition, the aminoacyl-tRNA biosynthesis pathway was also identified in patients with major depressive disorder (Yang et al., 2020a). The depression patients were found to have significant disturbances in amino acid metabolism, such as tryptophan and glutamic acid. The results indicated that the aminoacyl-tRNA biosynthesis pathway might be involved in the development of depression by affecting the synthesis of specific amino acids.

Besides, we studied the association between differential metabolites and gut virome using a co-occurrence network. Metabolites from the tryptophan metabolic pathway showed strong associations with viruses from *Microviridae*. Previous research demonstrated that improvements in the composition and metabolism of gut microbiota could affect peripheral tryptophan availability and central tryptophan levels, resulting in changes in central serotonin metabolism (Gao et al., 2020). Viruses may indirectly affect the pathophysiology of depression by interacting with bacteria, including altering their composition and metabolism. Previous studies have found increased lytic *Lactococcus* phages and decreased *Lactococcus* spp. in patients with Parkinson’s disease. The *Lactococcus* spp. is considered capable of producing microbiota-derived neurochemicals (Tetz et al., 2018). Another study reported an indirect link between phages and central nervous system (CNS) disease, revealing that *Lactobacillus phage φadh* was enriched in schizophrenia patients more than in controls, and the primary bacterial host was known to modulate intestinal permeability (Yolken et al., 2015). Therefore, the role of the gut virome in CNS diseases, such as depression, cannot be underestimated, but greater investment and research are required.

It should be mentioned that there are some limitations in this study: (i) Using CRS mouse model cannot fully reproduce the pathophysiology of depressed patients, the assumptions in our study need more experimental data to validate and more supports from clinical data; (ii) The small sample size is also an important limitation to be addressed in future research; (iii) Alterations in the gut bacteria of mice were not examined in this study. The interaction between gut bacteria and virome is also important to depression, which deserves attention in future studies.

In conclusion, we have demonstrated that CRS can result in gut virome dysregulation. Most of the differential gut virome in the mouse model of depression are *Microviridae*, and the differential metabolites are mainly enriched in the tryptophan metabolic pathway. The results also showed a strong association between the differential gut virome and neurotransmitters. We provide new insights into the role of the virome in depression pathogenesis. Research on gut virome and psychiatric diseases is still preliminary, while gut virome ecology research is primarily in the descriptive stages. In the future, it is more important to go beyond association researches in this field to study the causation of disease further.

## Data availability statement

The data presented in the study are deposited in the NCBI repository, accession number PRJNA877780.

## Ethics statement

The animal study was reviewed and approved by Animal Care Welfare Committee of the GuiZhou Medical University.

## Author contributions

JD, WW, and CL designed the study. JD wrote the manuscript. WW, JD, and XB performed the experiments and analyzed the data. WW, TJ, and CL reviewed the manuscript. All authors contributed to the article and approved the submitted version.

## Funding

This work was supported by the Natural Science Foundation Youth Fund Training Program of Affiliated Hospital of Guizhou Medical University (Grant no. gyfynsfc-2021-15).

## Conflict of interest

The authors declare that the research was conducted in the absence of any commercial or financial relationships that could be construed as a potential conflict of interest.

## Publisher’s note

All claims expressed in this article are solely those of the authors and do not necessarily represent those of their affiliated organizations, or those of the publisher, the editors and the reviewers. Any product that may be evaluated in this article, or claim that may be made by its manufacturer, is not guaranteed or endorsed by the publisher.

## Supplementary material

The Supplementary material for this article can be found online at: https://www.frontiersin.org/articles/10.3389/fmicb.2022.1046894/full#supplementary-material
