# Supplementary material for: Viral metagenomics combined with metabolomics reveals the role of gut viruses in mouse model of depression
Source: Front Microbiol. 2022 Nov 15;13:1046894. doi: 10.3389/fmicb.2022.1046894 (PMC9706091; doi:10.3389/fmicb.2022.1046894)
Supplement: Supplementary file 1 [file Data_Sheet_1.docx]

Supplementary Figures AND Tables


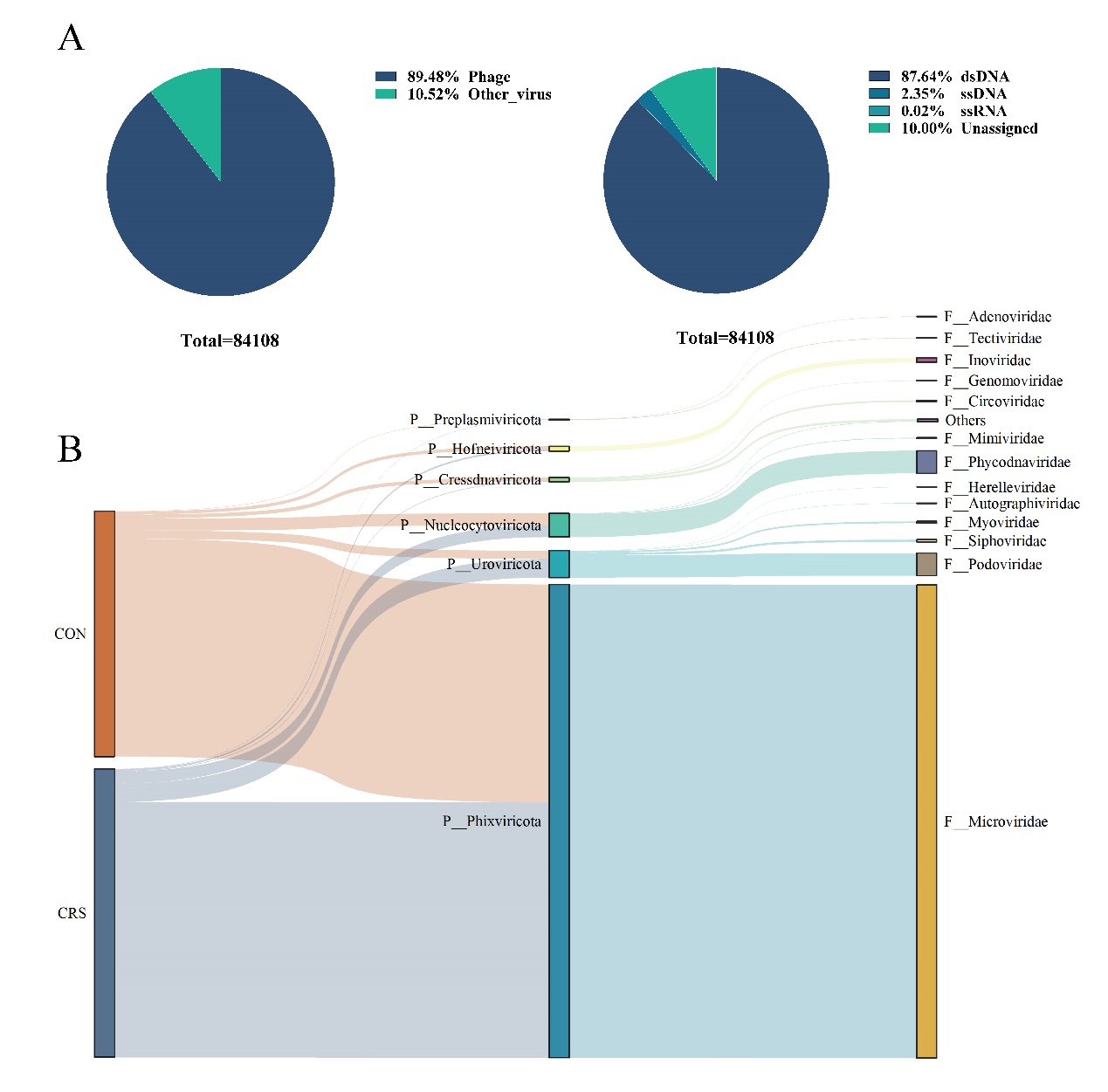


**Supplementary Figure 1**. Overview of gut virome sequencing and composition. (A) The ratio of phages and non-phages in the sequence is included in the analysis (left). The ratio of different types of viral genomes obtained from all samples (right). (B) Sankey diagram displaying the relationship between experiment samples and virome classification.


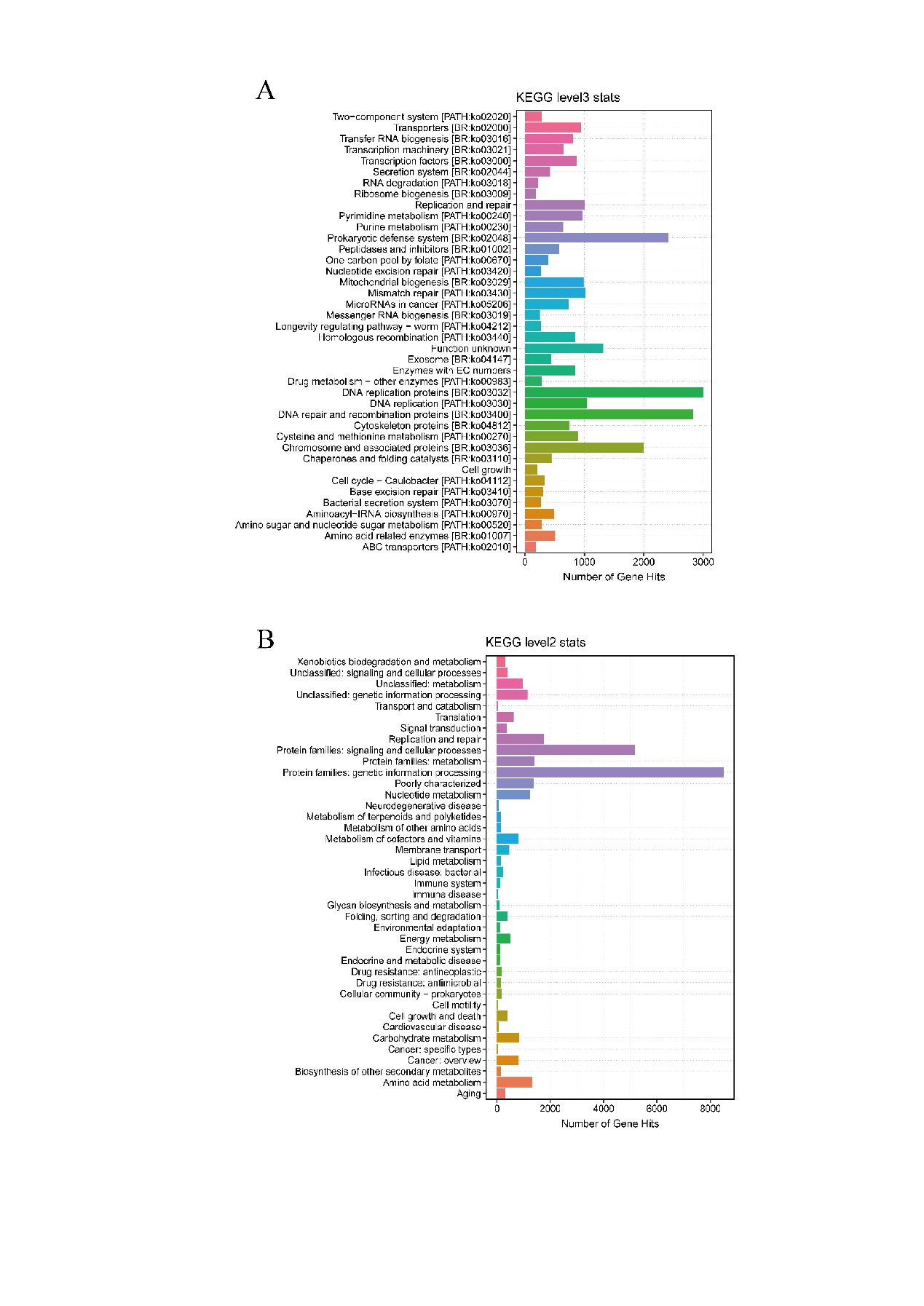


**Supplementary Figure 2**. According to KEGG annotation results of the viral genome, the histogram showed the number of genes on Levels 2 and 3 annotations for each sample.

**Supplementary Table 1. Baseline outcome measurements of SPT, FST and TST.**

|  | CON  (mean±SD) | CRS  (mean±SD) | t | *p* |
| --- | --- | --- | --- | --- |
| SPT | 0.854±0.054 | 0.890±0.042 | 1.489 | 0.160 |
| FST | 100.590±44.995 | 107.913±45.060 | 0.314 | 0.758 |
| TST | 143.414±33.315 | 131.169±65.657 | -0.464 | 0.652 |
